# Supplementary material for: Patterns of Intron Gain and Loss in Fungi
Source: PLoS Biol. 2004 Nov 30;2(12):e422. doi: 10.1371/journal.pbio.0020422 (PMC532390; doi:10.1371/journal.pbio.0020422)
Supplement: Table S1 — Also available at http://genes.mit.edu/NielsenEtAl/. (4.3 MB ZIP). [file pbio.0020422.st001.zip › NielsenEtAl/html/698.html]

AN2237.1.NCU05980.1.MG02309.1.FG08454.1


```
 CLUSTAL W (1.82) Multiple Sequence Alignments - Introns Inserted


Sequence 1: NCU05980.1	648 aa
Sequence 2: FG08454.1	675 aa
Sequence 3: AN2237.1	611 aa
Sequence 4: MG02309.1	618 aa
Alignment Length: 731 aa
Number Identitical Residues: 144 aa
Alignment Score (without introns) 8908


MG02309.1 	-----------MARLGLSLLAGAGALFGSALAQ-----FPP----APEGV----------
NCU05980.1	MWSPFNTRNGNGASPSLSRLLTLSTLAALAPLS--EAQFPP---YQAVGLKTVTSPSNPN
FG08454.1 	-----------MAAPHLLSLLYIRFLLAFAFVSSSSSQFLPGSDAKARNLTVVHSPANRD
AN2237.1  	-----------MPSL-TSVVGLSASLVGLVAGQY----FPP----TPEGVKVIHSKHQKG
          	            .      :     * . .  .     * *     . .:.   :  . .

MG02309.1 	------M0PGLCETTPG--VKSYSGYIHLPPGTLDVSGGLPQPYPINTFFWFFEARENPE
NCU05980.1	IKISYKE~PKDVCTTAFRRQKQYTGWVTVPG-----------EYPTHLFFWFVGAREP--
FG08454.1 	VTVSYKT~PEDVCSTAFDSQKQYTGWVSVPG-----------DYPTNLFFWFVEAREP--
AN2237.1  	VTISYKE0PEICETTPG--VKSYSGYVHLPPGTLNDLG-LDQQYPINTFFWFFESRNDPV
          	 . : .  *    :*.    *.*:*:: :* .: .  .   . ** : ****. :*: . 

MG02309.1 	NAPLSVWMNGGPGSSSMPGLFNENGPCFINPDSK---TTRLNPLSWNNKV1NMIYIDQ~P
NCU05980.1	TSALTMMLNGGPGSSSMFGLFAENGPCQVVEKGASRLETAAREWGWDRAS~NMLFVDQ~P
FG08454.1 	TDSLTIWLNGGPGSSSLYGFFTGNGPCEIVEKGLNEYETLVREWGWDRAS~NMLFIDQ0P
AN2237.1  	NAPLSIWMNGGPGSSSMIGLMQENGPCRVNIDSN---STELNPWSWNNYV~NMLYIDQ~P
          	. .*:: :********: *::  **** :  ..     *  .  .*:.   **:::** *

MG02309.1 	SQ0VGFSYDVLRNVTVDLFGGTRTLAPGSPIPPTNTTFRVGTLPSNNQNSTAVGSVNGAI
NCU05980.1	NH~VGFSYDTPTNGSLDLTTGMVSPTMQLPDNLPPSLFLNGTFSSNNIQNTANTTQNAAM
FG08454.1 	NQ~VGFSYDTPNNGTINFASNKVEVPPEANADYPDWVLRNGTFSSNNGDFTANTTETAAM
AN2237.1  	NQ~VGFSYDVPTNGTYNQLTGVRDVSGWSTVPEQNNTFYVGTFPSLNASATANTTENAAR
          	.: ******.  * : :   .    .           :  **:.* * . **  : ..* 

MG02309.1 	ALWHFFQIFLSEFPGYHP------KDDRFSIATQSYGGRYGPAIAGFFDAQNEKIRNGEF
NCU05980.1	AVYHLLQGFLSTFPEYVP---DKHSPLGVNLFTESYGGHYGPVFADTWQKENDKLSKRAM
FG08454.1 	AVWHMVQGFLTTFPQYQPSALSHNSSLGISLFAESYGGRYGPVFAETFVKQNARRQSGEL
AN2237.1  	ALWSFAQTWFTEFPAYKP------RDDRVSIWTESYGGRYGPSFSAFFQEQNEKIANGSL
          	*:: : * ::: ** * *          ..: ::****:*** ::  :  :* :  .  :

MG02309.1 	DNMAG----------------PKKIINLDTLLLVNACIERKVQWPSYPEQAFRNTYGIET
NCU05980.1	PLSRRQDDANNIQSRPPKAGSSNREIKLTSLGIMNGCIDDLVQGSRYVEMAINNTYGIKL
FG08454.1 	PYN------------------STIDVHLSSLGIVNGCVDMETQVPYYPTFAIDNTYNYKA
AN2237.1  	T-TPG----------------ESHYIHLDTLGIINGCIDLLVQAPSYPEIAYNNTYGIEA
          	                      .  ::* :* ::*.*::  .* . *   *  ***. : 

MG02309.1 	VSREVYESMTAAFNATG-GCREQIETCQALAKEYDPMQTGVNATVNRICQDSETWCTNNI
NCU05980.1	IDQATADAITSGFNAPDTGCKDLILACRQAQAALDPLDQGTDETVNQVCAYASLTCQQ-L
FG08454.1 	LSAGDAQFYQDKFTAEG-GCRDLLQKCATDASVGDPEGEGAEESINKACSDALNNCYD-I
AN2237.1  	INETIYEFAMNAWSKPG-GCKDQIVRCRQLAAEGDPRMYGNNKTVNAACQAADRYCSNNV
          	:.    :     :.  . **:: :  *       **   * : ::*  *  :   * :.:

MG02309.1 	RDPYIATSGRDYYDVTQLEPTMFPDPFVPGYLNQREVQLALGVPLNWTSSSSASSTAFRS
NCU05980.1	LGSVLA-SGANAYDIAHMGPDAFPDYHYLEYLNSRRVQEAIGSVVNYTDISPVVYQAFFQ
FG08454.1 	QDAYTS-SGRGVYDLAAASTDPFPPMTFLEYLNQESIQQAIGAPINYTLSNNEVGLEFQD
AN2237.1  	EGPYPLFSGRGYYDIAHFDPDPFPPPYYLGYLSQHWVQDALGVHINFTESIDSVYNAFGT
          	 ..    ** . **::   .  **      **... :* *:*  :*:*         *  

MG02309.1 	IGDYNR---PGWLEAMGNLLDKGKKVTLMYGDRDFACNWMGGEAASLAIPWRNQ------
NCU05980.1	TGDRAR---GGLIAKLASLLQRGVRIGLVYGDRDYICNWMGGEAVSLALADAMEDLDPKS
FG08454.1 	TGDQAR---GGNIQRLAALLNQGVRIALIYGDRDYICNWMGGEAVSQSLAQEAG-----S
AN2237.1  	VGDYARSDVRGYLEDLAYVLDSGIKVALVYGDRDYACPWNGGEQVSLQVDYSHA------
          	 **  *:.  * :  :. :*: * :: *:*****: * * *** .*  :           

MG02309.1 	---EKFAEAGYQPLRTNCTYDGGLVRQYSNLTFARVFQAGHAAPSYQQQTAYRIFNRALF
NCU05980.1	PYPTKFPQAGYENIQTNNREVGGVVRQFGNLSFSRIYQSGHFVPAYQPETAFRVFERIIG
FG08454.1 	EYSIRFPEAGYAPIIVNDSYIGGVVRQFGNLSFSRIYQAGHAMAAYQPETAFQVFARVIL
AN2237.1  	---DKFRAAGYAPLHTNSSYVGGSVRQYGNFSFTRVYQAGHEVPAYQPETAYEIFHRAMF
          	    :*  ***  : .*    ** ***:.*::*:*::*:**  .:** :**:.:* * : 

MG02309.1 	DKDVAGGLVDTATTPDFGTKGPASVRDVTNEKPPQYPYYCYILDTSS-CTDVQIAALRAG
NCU05980.1	GRSVATG--ARVNLRDFATRGPK-KADKQLKAPKDVSAKCFLRNLGSTCTQEQVMAILMG
FG08454.1 	GTSISTG--KDIDTTTYNTTGDA-NATHTDKLPDQPKPTCYIRNLRATCDDRAAELAAKD
AN2237.1  	NRDIATGKISTAKNSTYSTKGPSSTWNVTNTVPDSPEPTCYILSLSSYCTEEQTQSVANG
          	. .:: *         : * *  :        * .    *:: .  : * :        .

MG02309.1 	TAVIRDFIMVDPPYDPAAPRLAARDVRGMPTVDPDQRFY---------------------
NCU05980.1	EGVVINGVWYKEKKDWS--------PVTV-------------------------------
FG08454.1 	KGVVINGILYASMDDWPLQAETTEEPTTSTTTPSATSDMTGIYTATQTPEAAAAWNPPNS
AN2237.1  	TALIKDYIVVEESL----------------------------------------------
          	 .:: : :                                                    

MG02309.1 	---------------
NCU05980.1	---------------
FG08454.1 	GLVVAIIGIMVLHIV
AN2237.1  	---------------
          	
```
